# The continuous reaction time test for minimal hepatic encephalopathy validated by a randomized controlled multi-modal intervention—A pilot study

**DOI:** 10.1371/journal.pone.0185412

**Published:** 2017-10-11

**Authors:** M. M. Lauridsen, S. Mikkelsen, T. Svensson, J. Holm, C. Klüver, J. Gram, H. Vilstrup, O. B. Schaffalitzky de Muckadell

**Affiliations:** 1 Department of Gastroenterology, Hospital of South West Jutland, Esbjerg, Denmark; 2 Department for Gastrointestinal diseases, Odense University Hospital, Sdr. Boulevard 29, Odense C, Denmark; 3 Department of Endocrinology, Hospital of South West Jutland, Esbjerg, Denmark; 4 Department of Hepatology and Gastroenterology, Aarhus University Hospital, Aarhus, Denmark; Yonsei University College of Medicine, REPUBLIC OF KOREA

## Abstract

**Background:**

Minimal hepatic encephalopathy (MHE) is clinically undetectable and the diagnosis requires psychometric tests. However, a lack of clarity exists as to whether the tests are in fact able to detect changes in cognition.

**Aim:**

To examine if the continuous reaction time test (CRT) can detect changes in cognition with anti-HE intervention in patients with cirrhosis and without clinically manifest hepatic encephalopathy (HE).

**Methods:**

Firstly, we conducted a reproducibility analysis and secondly measured change in CRT induced by anti-HE treatment in a randomized controlled pilot study: We stratified 44 patients with liver cirrhosis and without clinically manifest HE according to a normal (n = 22) or abnormal (n = 22) CRT. Each stratum was then block randomized to receive multimodal anti-HE intervention (lactulose+branched-chain amino acids+rifaximin) or triple placebos for 3 months in a double-blinded fashion. The CRT is a simple PC-based test and the test result, the CRT index (normal threshold > 1.9), describes the patient’s stability of alertness during the 10–minute test. Our study outcome was the change in CRT index in each group at study exit. The portosystemic encephalopathy (PSE) test, a paper-and-pencil test battery (normal threshold above -5), was used as a comparator test according to international guidelines.

**Results:**

The patients with an abnormal CRT index who were randomized to receive the active intervention normalized or improved their CRT index (mean change 0.92 ± 0.29, p = 0.01). Additionally, their PSE improved (change 3.85 ± 1.83, p = 0.03). There was no such effect in any of the other study groups.

**Conclusion:**

In this cohort of patients with liver cirrhosis and no manifest HE, the CRT identified a group in whom cognition improved with intensive anti-HE intervention. This finding infers that the CRT can detect a response to treatment and might help in selecting patients for treatment.

## Introduction

The term minimal hepatic encephalopathy (MHE) denotes cognitive deficits in a patient with liver disease that are undetectable at clinical encounters. A MHE diagnosis, therefore, requires psychometric tests. The prevalence of MHE is very high in cirrhosis patients; i.e., present in approximately 50%[1, 2]. The condition is associated with severe loss of quality of life and markedly increased risks of clinically manifest HE that, in itself, is associated with increased mortality. Of note, MHE is usually treatable and treatment may improve all these untoward effects [3].

Despite the positive effects of diagnosing and treating MHE, only a few centres do so routinely[4]. One important hindrance is that it remains unclear if the necessary psychometric tests are clinically meaningful for predicting a positive treatment effect; i.e., knowledge is lacking about the test’s ability to predict cognitive improvements that are taken to be the basis of improved outcomes with treatment. Consequently, the basis on which patients can be selected for treatment is insufficient.

The underlying problem is that there is no gold standard for the diagnosis or grading of MHE. In specialist clinical centres, a few psychometric tests are used routinely in different combinations to diagnose MHE, but the test results are widely discordant in the individual patients [5–7]—although the group MHE prevalence with each one of the tests is similar. It remains uncertain how any of these tests should be validated for its ability to correctly identify patients with liver cirrhosis who may be expected to benefit from treatment.

We took advantage of the clinical reports on positive effects of MHE treatments to utilize anti-HE intervention as a tool for validation of a psychometric test: the continuous reaction time (CRT) test [3, 8, 9]. To have the strongest possible tool, we used the treatment principles reported to be efficacious in HE treatment in combination, viz. lactulose + branched amino acids + rifaximin. To avoid possible pitfalls; i.e., confounding due to the complex aetiology of MHE and its fluctuating time course, we used the intervention in a double blinded randomized prospective fashion in cirrhosis patients who did not present manifest HE.

The psychometric test we evaluated in this way, the CRT, is a simple PC-based performance test measuring the important cognitive ability of being able to deliver stable motor reactions to sensory stimuli—an ability of obvious importance for every-day living. In Scandinavia, this test is established for clinical routine use in the management of MHE. In the pilot study we present here, we also applied the portosystemic encephalopathy (PSE) test, a paper-and-pencil test recommended as an international comparator test for MHE studies.

Our hypothesis was that the CRT is able to identify patients with liver cirrhosis who improve cognition by anti-HE intervention. The aim of the study was to measure the CRT before and after such intervention vs. placebo.

## Patients and methods

### Patients

#### Reproducibility–cohort

Twenty-two patients with liver cirrhosis underwent repeated CRT testing to assess CRT reproducibility. They were included from 2 Danish sites (Odense University Hospital and Hospital of South West Jutland) during October 2012 without clinically manifest HE and were all tested twice before and twice after noon on two consecutive days reaching a total of 8 measurements per patient within 48 hours. Patients were aged 61 years (range 42–73 years), 18 were men, mean MELD score was 6.12 (range 6–16) and all had alcoholic cirrhosis. All patients were tested between 8.00 and 14.00 in an undisturbed location by one of 3 trained operators. All participants gave their informed written consent and The Regional Scientific Ethical Committee for Southern Denmark approved the study protocol (Protocol number S-20120196). In- and exclusion criteria cf. below.

#### The RCT-cohort

**Fig 1:**
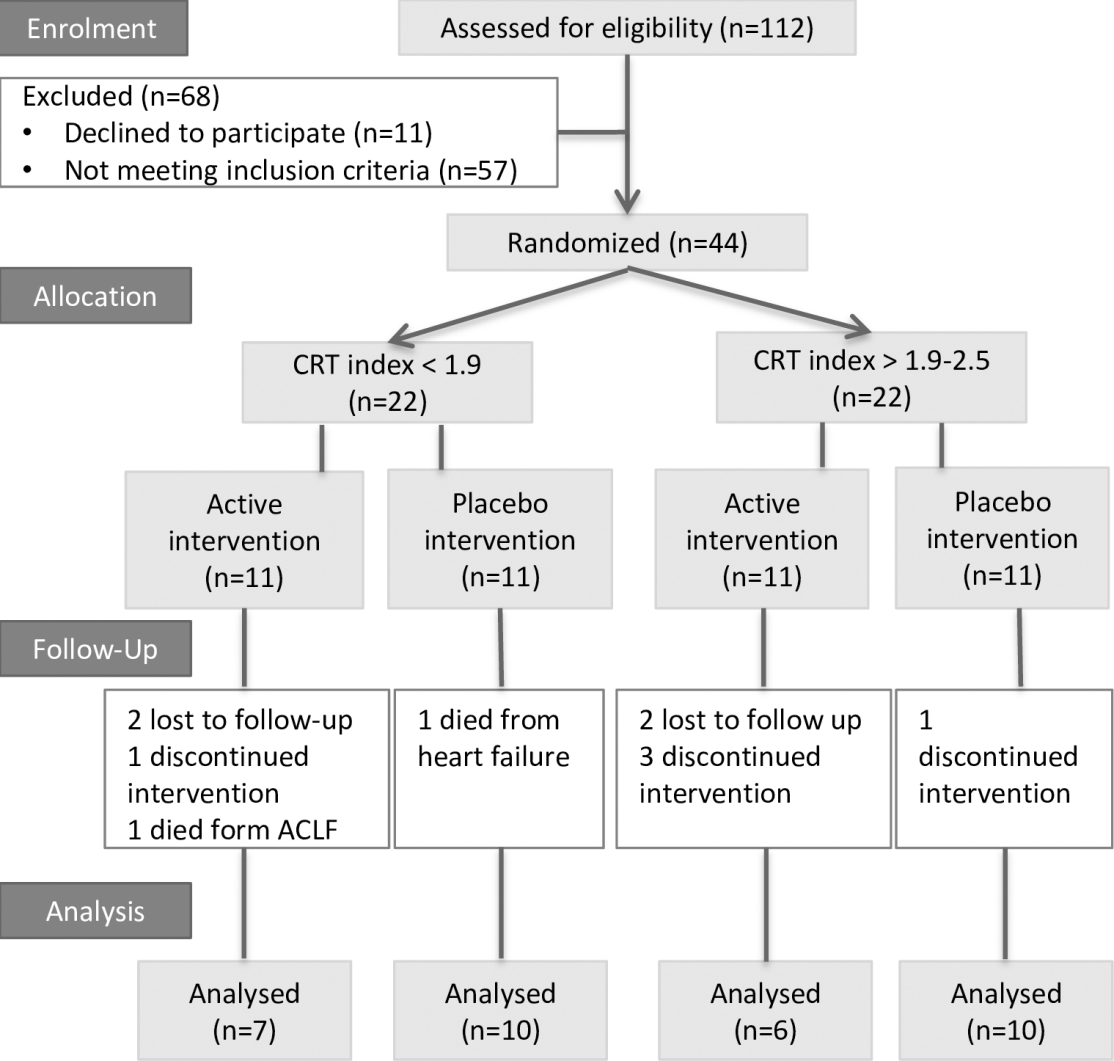
Patient flow through the study. A total of 44 patients were prospectively included and stratified according to a CRT index below or at 1.9 (abnormal) or above (normal). Patients were then block randomized into groups to receive either triple-active anti-HE intervention or triple placebo. We followed them for 3 months.

The inclusion criteria were age above 18 years, diagnosis of liver cirrhosis and absence of clinically manifest HE. From November 2013 to December 2014, we enrolled 44 such patients from the Hospital of Southwest Jutland (n = 23) and Odense University Hospital (n = 21; Fig 1). They were all cleared of manifest HE by experienced hepatologists. Liver cirrhosis was diagnosed by liver biopsy (19/44) or via unequivocal clinical, biochemical and ultrasonic findings. The exclusion criteria were HE of West Haven Grade 1 or more; organic brain disease; i.e., dementia or prior cerebrovascular disease; ongoing use of alcohol or psychoactive medication; severe hyponatremia (P-Na < 125 μmol/L), renal failure (P-creatinine > 150 μmol/L) or myxoedema; ongoing anti-HE treatment; acute bacterial infections, and sepsis or gastro-intestinal bleeding within a week of the test day. The included patients were, on average, aged 61.1 years (range 44–77 years), had 11.5 years (range 4–12 years) of formal education, 31 were men and alcohol was the dominant aetiology (37/44); 12 had previously manifest HE and 4 had a TIPS (2 in each treatment arm). All but 6 participants had previously participated in a cross-sectional study published previously (10), but upon inclusion in the study we present here they were psychometrically retested. All patients were tested with the two psychometric tests (CRT and PSE) at inclusion and at follow-up visits. Participants also completed a Sickness Impact Profile (SIP) questionnaire on their quality of life within the last 24 hours. Study entry characteristics of strata and randomized groups are summarized in Tables 1 and 2. The study time was 3 months and the patients were further followed up for an average of 10 months and episodes of manifest HE, hospital admissions, and deaths were registered. The descriptive entry CRT and PSE data were published earlier, apart from 6 new participants [10].

**Table 1:** Baseline characteristics for patients on active and placebo intervention.

	Active N = 22	Placebo N = 22
Age (years, mean and range)	59.6 (44–72)	61.1 (49–77)
Male/female	15/7	16/6
Cirrhosis aetiology Alc/Alc+HCV/HCV/unknown	19/0/1/2	18/2/0/2
White/blue collar	5/17	8/14
Education (years, mean and range)	11.2 (4–18)	11.7 (7–18)
MELD score (mean and range)	12.2 (6–20)	12.6 (7–24)
Child Pugh Score (mean and range)	8.0 (5–15)	7.4 (5–12)
Previous OHE	6/22	6/22
P-ammonia (μmol/L, mean and range)	40.0 (21–137)	39 (14–73)
Entry CRT index (mean and range)	1.7 (0.9–2.4)	1.8 (0.5–2.5)
Entry PHES (mean and range)	-8.0 (-15– -1)	-5.9 (-16– -2)
PHES and CRT agreement on MHE diagnosis	14/22	13/22

Abbreviations: CRT, continuous reaction time; PHES, portosystemic hepatic encephalopathy score; MELD, model for end stage liver disease; HE, hepatic encephalopathy; OHE, overt hepatic encephalopathy; MHE, minimal hepatic encephalopathy.

**Table 2:** Baseline characteristics for patients on active and placebo intervention stratified by continuous reaction time index (CRT index) at inclusion.

	CRT index < 1.9 (abnormal)		CRT index ≥ 1.9 (normal)	
	Active N = 11	Placebo N = 11	Active N = 11	Placebo N = 11
Age (years, mean and range)	60.3 (44–70)	64.2 (49–77)	59.0 (44–72)	57.9 (50–67)
Female/male	3/8	2/9	4/7	4/7
White/blue collar	3/8	5/6	2/9	3/8
Education (years, mean and range)	11.6 (8–18)	11.9 (7–16)	10.8 (4–14)	11.5 (8–18)
MELD score (mean and range)	12.0 (7–20)	13.9 (7–24)	12.55 (6–20)	11.45 (7–19)
Child Pugh Score (mean and range)	7.8 (6–16)	7.8 (5–12)	8.4(5–12)	6.9 (5–9)
Previous OHE	2	4	5	2
Entry CRT index (mean and range)	1.3 (0.9–1.7)	1.4 (0.5–1.8)	2.1 (1.9–2.4)	2.1 (1.9–2.6)
Entry PHES (mean and range)	-8.5 (-15– -5)	-6.7 (-16– -2)	-7.6 (-13– -1)	-5.1 (-13– -1)
PHES and CRT agreement on MHE diagnosis	11/11	8/11	3/11	5/11
P-ammonia (μmol/L, mean and range)	57 (25–137)	34 (14–65)	43 (22–41)	29 (15–73)

Abbreviations: CRT, continuous reaction time; PHES, portosystemic hepatic encephalopathy score; MELD, model for end stage liver disease; HE, hepatic encephalopathy; OHE, overt hepatic encephalopathy; MHE, minimal hepatic encephalopathy.

### Methods

#### The continuous reaction time test (CRT)

The test is PC-based and requires a standard laptop, simple software, headphones, and a handheld trigger button (EKHO, www.Bitmatic.com, Aarhus, Denmark). After a 2–minute instruction, the patient is left alone and undisturbed to complete the 10-minute test during which the patient responds to 150 sound stimuli (beeps at 500 Hz and 80 dB) by pressing a button. They are delivered at random intervals from 2 to 6 seconds and the patient is instructed to press the button as soon the beep is heard. The software registers the response times and calculates the CRT index (the ratio: 50 percentile/[90 minus 10 percentile]). The method measures and combines motor reaction speed, sustained attention, and inhibitory control, which are all key abilities in daily life functioning [11].

The group change in CRT index was the primary study outcome. The CRT index is not affected by age, gender, educational level or intelligence [12–15]. The established threshold for normal values is a CRT index of 1.9. An index below 1.9 reflects poor reaction time *stability* and may indicate MHE in patients with cirrhosis. The threshold is based on prior studies designed to discriminate between HE patients, controls, and patients with organic brain damage with a sensitivity and specificity above 90%[16].

#### The portosystemic encephalopathy test (PSE)

This was a secondary study outcome. The test is a paper-and-pencil test consisting of 5 sub-tests: Digit Symbol test (DST), Number Connection Test A (NCT-A), Number Connection Test B (NCT-B), Serial Dotting Test (SDOT), and Line Tracing Test (LTT, time and errors). Completing all sub-tests takes approximately 15 minutes and the presence of staff for guidance and timing is required. Calculation of the final test result using age-adjusted norms takes another 5 minutes. The test score is calculated from the time (seconds) spent on each sub-test, which is converted into a score that ranges between -3 and 1. The LTT is converted into 2 scores—a time score and an error score. A score of 0 is given for a performance within +/- 1 SD. The total test score, the portosystemic hepatic encephalopathy score (PHES), is the sum of the scores. A result below -4 is abnormal and may indicate MHE in liver patients. Danish norm data (personal communication) were applied. The test measures attention, working speed and accuracy, visual orientation, and visuo-spatial construction [5]. Multiple versions of the test battery are available to prevent learning effects with repeated testing [17].

## Interventions

After initial testing and stratification according to the CRT index, the patients were block randomized into two groups to receive either triple-active anti-HE intervention or triple placebo for 3 months. The patients randomized to active intervention received 66.7% lactulose syrup (Medilax, up to 75 mL/day), branched-chain amino acids (Bramino, powder formulation, 17 g/day) and rifaximin (Xifaxan, tablets, 550 mg b.i.d.). The triple placebo regimen consisted of placebo to lactulose (50% glucose syrup), placebo to branched-chain amino acids (alanine powder) and placebo to rifaximin (inactive tablets identical to the active tablets). Patients received oral and written information about possible adverse effects of the medications. The effect of the interventions was measured after study end. A protocol summary is available in S1 Text and the full protocol in S2 Text.

## Randomization

The Odense University Hospital Pharmacy boxed active and placebo medications in numbered packages and intervention assignment was obtained from a computer-generated randomization list in blocks of 22. All items were labelled with only the patient and study number (numbers 1–44). Boxes numbered 1–22 contained 11 sets of active medicine and 11 sets of placebo medicine in random sequence and were consecutively assigned to the stratum of participants with a CRT index < 1.9. The boxes numbered 23–44 likewise contained the same sets as the first boxes and were sequentially assigned to the stratum of participants with a CRT index greater than or equal to 1.9. In this way, all the patients, including those with a CRT index within the normal range (up to a CRT index of 2.5), randomly received active intervention or placebos. The study participants and all staff were blinded until all data were collected.

## Ethics

There were no reported serious adverse effects for any of the components of the active and placebo interventions. We considered it ethically acceptable to administer the interventions to both cirrhosis patients with and without psychometric signs of MHE and without the aim of documenting a clinical outcome benefit. The potential benefit to future patients lies in the prospective of improving the possibilities of identifying such patients who can be expected to improve their cognition by treatment. The study principle, methods and protocol complied with the 1975 Declaration of Helsinki and was approved by the Danish National Committee on Health Research Ethics and registered at clinicaltrial.gov (Identifier: NCT01773538). All participating patients gave their informed written consent.

## Statistical analysis

RCT data and reproducibility data are available in S1 Data and S2 Data. In the reproducibility analysis we assessed the between-subject variations by calculating group means and scatter (= 2SD). Within subject reproducibility was assessed by the mean intra-subject scatter of CRTindices.

For the RCT our hypothesis was that patients randomized to active intervention would obtain an improvement in the CRT index significantly greater than that resulting from the placebo intervention. Accordingly, our primary outcome was the difference in mean CRT index change between active and placebo groups. We wanted a type I error of 5% and a type II error of 15% (power 85%). The expected dropout rate was set at 20%. We assumed that the standard deviation of repeated measurements is a reasonable estimate of the unknown standard deviation of *changes* in CRT index and the desired effect size was set at 0.68. We found that 6 patients were needed per group. This number was increased to 9 as the true SD of changes in CRT index most likely is larger. As we ultimately decided to have 2 stratae in each group we included 36 patients plus 8 to take drop outs in to account i.e. a total of 44–22 in each treatment arm [18]. The CRT index data showed Gaussian distribution as evaluated by the histogram and using the Shapiro-Wilk test we found a non-significant p-value (p = 0.41) indicating that the sample came from a normally distributed population [19]. Student’s t-test was therefore used to test for intra- and inter-group differences in CRT index. For the PHES Wilcoxon rank-sum test was used. We used one-sided p-values in the comparison of pre and post treatment results in the groups receiving active intervention as we assumed to know the direction of the CRT index and PHES change. In all other cases two-sided p-values were used. Difference between proportions was assessed using Fisher’s exact test. Statistical analysis was performed using Prism 6 GraphPad for Mac OS X.

## Results

### Reproducibility

**Fig 2:**
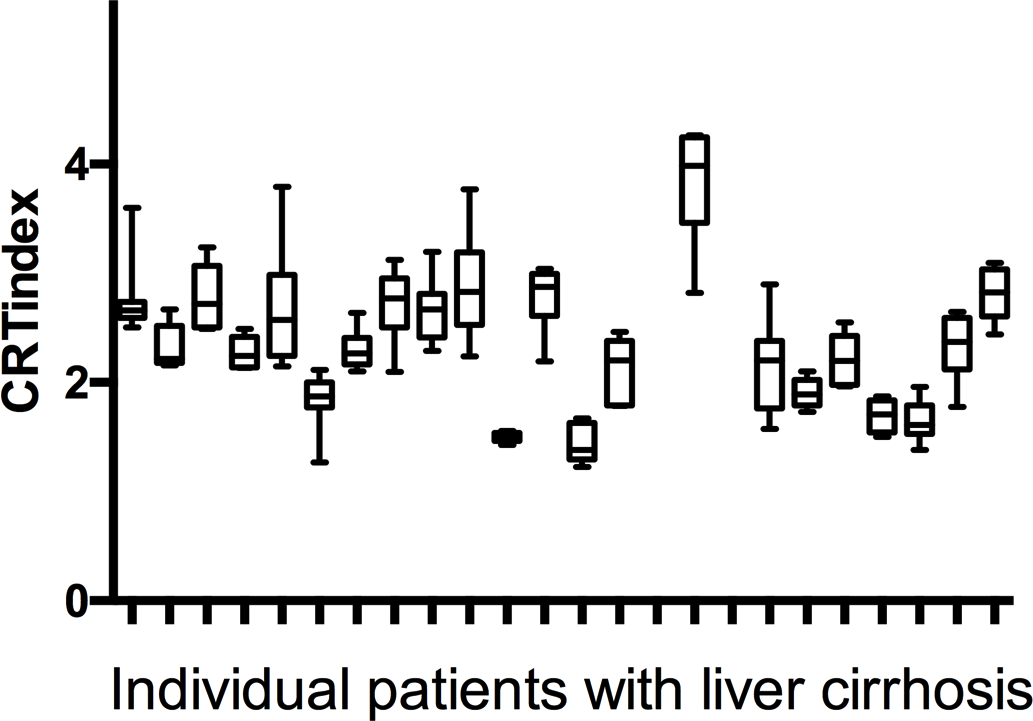
Data from 22 patients with liver cirrhosis who all underwent 8 repeated continuous reaction time (CRT) measurements: 4 measurements on to consecutive days.

Results are displayed in Fig 2. Within subject SD showed a negligible decrease with increasing age i.e. decreasing with approximately 0.01 per decade. We chose not to take age in to account and found that in cirrhosis patients the group mean CRTindex was 2.35 (SD 0.61, 95%CI 2.3–2.4, range 0.67–3.87). The within-subject mean SD was 0.34. Accordingly, a CRTindex change of more than 0.68 (2SD) in a cirrhosis patient can be taken to reflect a significant change in CRTindex. Time of day did not influence the CRTindex or mean in either group.

### RCT

Seventeen patients with an abnormal CRT index below 1.9 (7 actively treated, 10 placebo) and 16 with a normal CRT index ≥1.9 (6 actively treated, 10 placebo) completed the 3-month study (Fig 1).

#### Effects of the anti–HE intervention on the CRT index according to active vs. placebo

Baseline characteristics are shown in Table 1. The mean CRT index improved from 1.7 to 2.2 (p = 0.02) in the patients on active intervention and remained stable at 1.8 in the placebo group (Table 3). There was a marginal difference in the exit CRT index values between the active and placebo groups (2.2 vs. 1.8, p = 0.07). There was also a marginal difference in the mean CRT index change between the patients on active and placebo intervention (a mean CRT index change of 0.50±0.20 vs. 0.13±0.12, p = 0.06). The mean PHES improved from -8.0 to -5.0 in the patients on active intervention (p = 0.02). There was no difference in exit PHES between the active and placebo group (-5.0 vs. -5.5, p = 0.40) but the mean PHES change was larger in the active group (3.9±0.9 vs. -0.8±0.8, p = 0.02).

**Table 3:** Psychometric results and changes at the 3-month follow up in the active and placebo intervention groups.

	Active N = 13	Placebo N = 20	P
Psychometry at 3-month follow-up			
CRT index (mean and range)	2.2 (1.6–3.4)	1.8 (0.9–3.3)	0.07
ΔCRT index (mean change and SEM)	0.50 ± 0.20	0.13 ± 0.12	0.06
Normalized CRT index	5/7 (71%)	3/10 (30%)	0.41
PHES at 3 months (mean and range)	-5.0 (-16– –1)	-5.5 (-15–1)	0.40
ΔPHES (mean change and SEM)	3.9 ± 0.9	0.8 ± 0.8	**0.02**
Normalized PHES	6/7 (85%)	3/5 (60%)	1.0

Abbreviations: CRT, continuous reaction time; PHES, portosystemic hepatic encephalopathy score

#### Effects of the anti–HE intervention on the CRT index according to the entry CRT index

The stratum with an abnormal baseline CRT index: Baseline characteristics are displayed in Table 2 and results after 3 months of intervention in Table 4. The patients with an abnormal entry CRT index and randomized to the active intervention improved their CRT index from 1.3 to 2.3 (p = 0.01) and, thus as a group, (5 of 7 patients) normalized their CRT index. No such improvement was seen in the corresponding patients randomized to the placebo intervention (1.4 to 1.6, p = 0.15) who, as a group, continued to have an abnormal CRT index (Table 4). Likewise, the mean CRT index change was larger in the patients on active intervention (0.92 ± 0.29, 95%CI -0.08–0.68) vs. those on placebos (0.25 ± 0.18, 95%CI -0.27–1.56; active vs. placebo p = 0.03; Table 4). The PHES also improved in the patients with an abnormal entry CRT index on active intervention (a mean entry PHES -9.8 vs. a mean exit value -6.7, p = 0.003). Again, the mean PHES change was larger in the active treated vs. placebo groups (4.6 ± 1.3, 95%CI 1.2–7.9 vs. -0.20±0.13, 95%CI -3.2–2.7, p = 0.03; Table 4) and in all cases concomitant with the change in CRT.

**Table 4:** Main results in the strata with CRT index <1.9 (abnormal) and 1.9 (normal). For continuous variables, the mean and range or standard error of the mean are given.

	CRT index < 1.9 (abnormal)			CRT index ≥ 1.9 (normal)		
3-month follow-up	Active n = 7	Placebo n = 10	P	Active n = 6	Placebo n = 10	P
CRT index (mean and range)	2.3 (1.6–3.4)	1.6 (0.9–2.5)	**0.02**	2.0 (1.7–2.7)	2.2 (1.4–3.3)	0.30
ΔCRT index (mean change and SEM)	0.92 ± 0.29	0.25 ± 0.18	**0.03**	0.07 ± 0.07	0.06 ± 0.19	0.34
Normalized CRT index	5/7	3/10	0.12	-	-	-
PHES at 3 months (mean and range)	-3.6 (-9– -0)	-7.6 (-15–0)	0.03	-6.7 (-16–3)	-3.4 (-11–1)	0.74
ΔPHES (mean change and SEM)	4.6 ± 1.37	-0.20 ± 1.32	**0.03**	3.1 ± 1.35	1.7 ± 0.93	0.14
Normalized PHES	4/11	1/8	0.34	2/8	2/6	1.0
P-ammonia (μmol/L, mean and range)	44 (16–108)	41 (15–59)	0.82	39 (26–65)	40 (16–67)	0.96

Abbreviations: CRT, continuous reaction time; PHES, portosystemic hepatic encephalopathy score.

The stratum with a normal baseline CRT index: No change from the baseline CRT index was found in the active or the placebo group. The PSE test also did not change. There was no difference in CRT and PSE changes (Table 4).

Ammonia and manifest HE episodes: The P-ammonia concentration decreased on average by 22% (from a mean of 57 to 44 μmol/L, p = 0.05) in the group with an abnormal entry CRT index and randomized to active intervention, but such an effect was not observed in any other group. No significant or systematic change in Child Pugh, MELD, or SIP score was observed in either strata or group. CRP remained unchanged in all groups.

During the post–study follow-up, 2 out of 13 in the group receiving active intervention and 4 out of 20 in the placebo group experienced an episode of manifest HE. In the active group, 1 patient died (acute-on-chronic liver failure) while 5 patients in the placebo group died (2 of infection, 1 of heart failure, 1 of variceal bleed, and 1 postoperatively after a case of ileus; p = 0.36).

Post hoc analysis of psychometric effects according to entry PSE: The patients were primarily stratified according to their CRT index, so the analysis according to PSE is an exploratory secondary analysis. It is based on 25 patients with an abnormal entry PSE, 13 of them receiving the active intervention and 12 of them receiving the placebos. Those on the active intervention improved their CRT index from 1.6±0.4 to 2.2±0.5 (p = 0.02) and PHES from -8.9±3.2 to -5.0±4.4 (p = 0.0014). No improvement in PHES or CRT index was observed in the placebo group (-9.0±3.6 to -7.6±4.2, p = 0.22 and 1.7±0.5 to 1.8±0.5, p = 0.87).

#### Dropouts

The overall dropout rate was 25% (n = 11) and thus 5% larger than expected. Nine (40%) dropped out in the group randomized to active invention and two (9%) in the placebo group (p = 0.08). Patient characteristics and reasons for dropout are listed in Table 5. A post hoc sensitivity analysis considering all dropouts showed that the results would have been the same if none had dropped out: in a best-case scenario all dropouts would have experienced improvement similar to that of the patients on active intervention and these patients still improved the most (p = 0.04). In the worst-case scenario, all dropouts would have improved corresponding to the average placebo CRT index change and, in this case, the difference between the groups was still significant (p = 0.03).

**Table 5:** Dropout characteristics.

	Entry CRT	Entry PHES	MELD score	Child Pugh Score	Reason for drop out
Active anti HE treatment					
1	2.1	-5	17	12C	OHE
2	2.2	-11	15	9B	Orthopaedic hospital admission
3	1.7	-15	17	11C	Dead
4	0.9	-5	9	7B	Adverse effects
5	2.3	-2	6	5A	Adverse effects
6	2.1	-4	17	7B	No reason given
7	1.1	-7	14	6A	No reason given
8	1.5	-7	11	8B	No reason given
9	1.9	3	14	9B	No reason given
Placebo					
1	1.8	0	24	9B	Died from previously diagnosed heart failure
2	1.9	-4	10	6A	OHE

## Discussion

The purpose of this pilot study was to evaluate if the CRT test is able to detect and predict an effect of anti-HE-treatment. The central finding was that the cirrhosis patients without manifest HE and with an abnormal CRT index normalized or improved their psychometric status i.e. CRT index (and PHES) with three months of triple-active anti-HE intervention—this did not occur in any other study group. The clinical implication of this finding it is that the CRT test might be useful in detecting an effect of anti-HE treatment (and useful in identifying cirrhosis patients who will benefit cognitively from anti-HE treatment). The test thus could be used both in the daily clinic and as an outcome measure in future scientific studies.

The methodological novelty of our study is that we used the effects of reportedly efficient therapeutic interventions in a randomized controlled fashion as a means to validate the meaningfulness of the CRT method in the management of MHE. Our primary outcome was the changes in psychometry i.e. the CRT index whereas we did *not* primarily aim to test the intervention effect. To secure the presence or non-presence of the desired psychometric signal, we applied the expectedly most powerful anti-HE intervention viz. the multi-modal triple modality. It is possible that we would have reached the same result with lactulose as a mono-intervention as there is now evidence that lactulose does ameliorate MHE, albeit the diagnostic methods vary among studies [3]. The current HE management guidelines do not recommend routine anti-MHE treatment likely due to uncertainty regarding which patients who would benefit from it [20]. The results for our study may improve this lack of decision basis when it comes to the CRT test; and demonstrate a methodological approach that could be useful in a similar validation of other psychometric tests for MHE. The ability of the CRT test to predict a treatment response, however, still does not solve the basic issue of establishing a gold standard for the diagnosis of MHE.

Additionally, the comparator measure, the PSE, improved in the group randomized to active intervention as whole (Table 3) and the same effect was observed in the subgroup with an abnormal entry CRT index. Furthermore, in the secondary analysis, the patients with an abnormal entry PSE who were on active intervention also improved their CRT index and PHES. Although this was not a defined study outcome, it may illustrate that the PSE results, despite being somewhat discordant from the CRT results, may also be of use in identifying patients who would benefit from anti-HE intervention. Still, the PSE did not normalize on a group level, unlike the CRT index [10]. These results may be used to support the basic concept of the clinical utility of psychometry.

No other psychometric validation study within this research field is based on the same approach. It is noteworthy that the patients with an abnormal CRT index who received the placebo intervention tended to improve their CRT index—only less so than the patients on the active intervention (by average 0.25 vs. 0.92). This illustrates the time-dependence of the cognitive disturbances cause by MHE and emphasizes the necessity for randomized intervention for evaluation of the test. As could be expected, there were no such temporal dynamics in the patients with a normal entry CRT index.

Our salient result was obtained in the small group of patients with an abnormal entry CRT index and randomized to active intervention. However, it was statistically significant and based on the *a priori* assumptions after which the study was dimensioned. Its robustness is supported by the fact that the outcome effect was more than threefold larger than the intra-individual day-to-day CRT index variability that we measured. It also supports the CRT index improvements being paralleled by the PSE test. The mechanistic role of ammonia is equivocal but, if anything, it is in support of our results that the group with the improvement in CRT index was the also the one in which ammonia decreased. We do, however, have a weakness due to the small sample sizes of the groups where no significant results were obtained. In these, the lack of signal might be caused by chance, although the negative results can be taken to have the power assumed in the *a priori* power calculation since there were only few dropouts in these groups.

The total number of dropouts was higher than assumed, but the sensitivity analyses still showed this did not threaten the study conclusions. The stratification after the entry CRT index did not predict dropout, but randomization to the active intervention did predict dropout. As usual, the most common complaint was bowel discomfort, but for those who chose to continue in the protocol, the complaints diminished within 7–10 days. No serious adverse event was reported.

We did not observe any change in disease related quality of life in either group or strata. This contrasted with prior studies [21–23] and the discrepancy was most likely related to our small study groups.

In summary, we showed that, in patients with cirrhosis and without manifest HE, an abnormal CRT index seemed to identify those patients who proved to cognitively normalize or improve with multimodal anti-HE intervention. Our findings needs confirmation in larger studies but indicate that the CRT test may be useful in selecting cirrhosis patients for treatment for MHE—a frequent and debilitating complication. Further, our methodological approach could be useful in a similar validation of other psychometric tests.

## Supporting information

S1 Text Protocol summary. (PDF)

S2 Text Full protocol. (PDF)

S1 Data Main RCT data. (XLS)

S2 Data All reproducibility data. (XLSX)

S1 Table Consort 2010 Checklist. (DOC)
