# Supplementary material for: The continuous reaction time test for minimal hepatic encephalopathy validated by a randomized controlled multi-modal intervention—A pilot study
Source: PLoS One. 2017 Oct 11;12(10):e0185412. doi: 10.1371/journal.pone.0185412 (PMC5636096; doi:10.1371/journal.pone.0185412)
Supplement: S1 Text — (PDF) [file pone.0185412.s001.pdf]

PhD Student Mette Munk Lauridsen
Hospital of South West Jutland, Denmark

PhD project

Diagnosis of Minimal Hepatic Encephalopathy - a descriptive and experimental human patho-etiological study focused on the use of continuous reaction time measurements

Protocol Summary

This PhD project is carried out in collaboration between Hospital of South West Jutland, Esbjerg, Denmark, Odense University Hospital, Odense, Denmark and Aarhus University Hospital, Aarhus, Denmark.

Key researcher: MD, PhD student Mette Munk Lauridsen, Hospital of South West Jutland, Finsensgade 35, 6700 Esbjerg, Denmark. Mobile phone: +4561661192, mettelauridsen@gmail.com

Supervisor: Professor Ove Schaffalitzky De Muckadell, Odense University Hospital, Department of gastroenterology.

Other supervisors: PhD, chief physician Jeppe Gram, Hospital of South West Jutland, Esbjerg and Professor Hendrik Vilstrup, Department of hepatology and gastroenterology, Aarhus University Hospital.

Test site: Department of hepatology and gastroenterology, Hospital of South West Jutland, Esbjerg, Finsensgade 35, 6700 Esbjerg and Odense University Hospital, Department of gastroenterology, Sdr. Boulevard 29, 5000 Odense C,

Background

Liver cirrhosis is often complicated by minimal hepatic encephalopathy (MHE). Patients with MHE have poor quality of life; and a higher risk of falls, traffic accidents, and episodes of overt hepatic encephalopathy as compared to patients with no encephalopathy. MHE is treatable in most cases. Lactulose, branched chain amino acids (BCAA) and rifaximin are currently the available treatment options. By definition MHE is unrecognizable at physical evaluation and must be diagnosed via sensitive psychometric methods. There are several available on the marked. Our project looks at the possibility of diagnosing hepatic encephalopathy by means of

a computerized method called CRT – continuous reaction time measurements. The CRT test is simple and quick to perform and this is a major advantage compared to traditional psychometric methods preformed with paper and pencil. The CRT test has been the preferred screening tool for MHE for more than 30 years in Denmark when MHE wasn't even perceived as a diagnostic entity. The CRT test therefore it needs new and more thorough validation for the use as a diagnostic and monitoring tool for MHE.

In our study we use the portosystemic encephalopathy test (PSE test) as a comparator test. We do so because the PSE test is the most widely used psychometric test for MHE and endorsed as a common comparator test by ISHEN (International Society for the study of Hepatic Encephalopathy and Nitrogen Metabolism). This paper-pencil test battery consists of 5 sub tests. Age–adjusted normal values are used to score a patient's performance in the test. The overall score is termed the PHES and a value below -4 is abnormal.

Objective

The aim of this PhD project is to investigate whether continuous reaction time measurements (CRT) are suitable as a screening and monitoring tool for minimal hepatic encephalopati.

Method

CRT test: The CRT test is a 10-minute computerized test during which a patient's reaction time to auditory stimulus is measured 150 times. The intra–individual reaction time variation is the key test result and is calculated as 50 percentile (sec)/90-10 percentile (sec) and termed the CRT index. A CRT index below 1.9 is indicative of brain dysfunction i.e. hepatic encephalopathy in patients with liver cirrhosis.

PSE test: This paper-pencil test battery consists of 5 sub tests. Age–adjusted normal values are used to score a patient's performance in the test. The overall score is termed the PHES and a value below -4 is abnormal.

Study design

Sub-protocol 1: As a part of this PhD protocol 100 healthy individuals and 60 with chronic disease (chronic obstructive pulmonary disease, heart failure, kidney failure, diabetes II) will

be tested using the CRT and PSE tests. This is to determine the normal range for the PSE test in the Danish population.

Sub-protocol 2: A total of 120 patients with liver cirrhosis from two Danish hospitals will be examined with both CRT and with the test that is the closest we get to a gold standard, namely portosystemic encephalopathy test (PSE). We wish to examine if the CRT test agrees with the PSE test, which may be to time consuming to perform in everyday clinical practice, and with quality of life scores. The relationship between the CRT and PSE test and various blood tests and the Charlson co-morbidity score will also be examined.

Sub-protocol 3: 44 of the 120 included patients with CRT index up to 2.5 will be randomized in a balanced fashion to treatment with lactulose, rifaximin and branched chain amino acids (BCAA) or placebo lactulose, rifaximin and BCAA based on their entry CRT index: 22 patients with CRT index below 1.9 will be randomized to active or placebo intervention and 22 patients with CRT index 1.9–2.5 will likewise be randomized to active or placebo intervention. This is to evaluate whether the CRT method is able to detect a response to treatment and assess if the established CRT index cut off point at 1.9 is appropriate. A secondary aim will be to see if changes in psychometric tests (PSE and CRT) are in accordance with quality of life scores and will predict development of overt hepatic encephalopathy. The power calculation was done based on knowledge about CRT index reproducibility and a wish to detect index changes larger than 0.30 at a 5% significance level and a power of 85%.

Perspective: CRT method should, if it proves good enough, continue to be the Danish test of choice and hopefully be more widely used in our country. The validation of tests for the diagnosis of covert hepatic encephalopathy will give cirrhotic patients with covert hepatic encephalopathy and reduced quality of life the best opportunity to be diagnosed and offered appropriate treatment. If the CRT method is not able to identify a population that benefits from anti-encephalopathy treatment other screening and monitoring tests should be used.

Test population

The goal is to include 120 patients with liver cirrhosis, confirmed by biopsy or via ultra sound or CT scan along with relevant biochemical markers in combination with a relevant history.

Inclusion criteria

1) Age> 18

2) Liver cirrhosis confirmed by biopsy or appropriate clinic and biochemistry, and imaging.

3) Written informed consent

4) Speak and understand Danish

By inclusion of cirrhotic patients and at follow-up after 3 months the following tests will be conducted:

1) The CRT test

2) Quality of life scores (SIP and SF-36)

3) Test PES

4) MMSE Test

5) Blood tests: haemoglobin, S-ethanol, bilirubin, ALT, BASP, PK, creatinine, albumin, sodium, CRP, TSH, magnesium, zinc, potassium, ammonium, arterial addition, a blood sample is to be stored in the bio-bank for later analysis

6) Height and weight measured

7) Charlson co-morbidity score

8) If patients have a cohabiting partner they will be questioned about their partner/the patients daily functioning via a standardized questionnaire (IQCODE)

Healthy controls will be examined with:

1) The CRT test

2) PES-test

Exclusion criteria for cirrhotic patients and control persons:

1) Clinical manifest hepatic encephalopathy

2) Consumption of psychoactive substances within 6 days of test

3) Organic brain disease (i.e. prior stroke, dementia)

4) Hypothyroidism

5) Renal failure (creatinine> 150 mg / dL)

6) Hyponatremia (Na <125 mmol / L)

7) Sepsis or bleeding within one week prior to testing.

8) Serious sleep disorders

9) Current treatment with lactulose, rifaximin or BCAA

Publication

The results from the project are sought published in international scientific journals in hepatology or neurology. Both positive and negative test results will be published.

Ethical considerations

The benefits of new knowledge on hepatic encephalopathy this study will provide are estimated to exceed the test population's disadvantages. The test participant will have done a blood sample, which is also a part of the daily monitoring of patients with cirrhosis of the liver and poses no further disadvantage. Participation in the project will require approximately 2 hours of the patients' time at each examination. The participants may benefit from the treatment they are offered. Patients will not be remunerated for their participation in the project.

Treatment with lactulose and rifaximin is associated with the risk of side effects. But in the literature no serious side effects are reported for the drugs in question. If significant side effects occur, the treatment course is stopped or downgraded to perceive fewer preparations.

Ethical considerations related to randomization

Some patients are offered treatment and some placebo. Screening for and treatment of minimal hepatic encephalopathy is not done routinely in Danish hospitals and there is still no

national guidelines saying that we should do this. The patients that are offered placebo will therefore be offered what is "standard of care" in most hospitals in the country and this is therefore considered to be ethically justifiable. Should an untreated patient in the study developed overt HE the usual treatment for this will of course be initiated.

Recruitment of participants

At outpatient contacts regarding diagnosis and treatment of liver cirrhosis patients are contacted and informed of the opportunity to participate in a scientific research project. It may also be necessary to telephone the patients to arrange a meeting where the patient is informed of the opportunity to participate in the project. In addition to oral information, written information about the project and the booklet "Your Rights as a test subject in a biomedical research" will be give to each patient. The project can begin immediately after informed consent was given. If any doubt or question the patient can at any time contact the project manager who will be the contact person.
