# Supplementary material for: The continuous reaction time test for minimal hepatic encephalopathy validated by a randomized controlled multi-modal intervention—A pilot study
Source: PLoS One. 2017 Oct 11;12(10):e0185412. doi: 10.1371/journal.pone.0185412 (PMC5636096; doi:10.1371/journal.pone.0185412)
Supplement: S2 Text — (PDF) [file pone.0185412.s002.pdf]

Videnskabelig Protokol

Ph.d projekt

Diagnostik af Minimal Hepatisk Encephalopati

- et deskriptivt og eksperimentelt human pato-ætiologisk studie med fokus på anvendelse af kontinuerte reaktionstidsmålinger

Projektansvarlig

Hoveduddannelseslæge Mette Munk Lauridsen

Medicinsk Gastroenterologisk Afdeling, Sydvestjysk Sygehus

Finsensgade 35, 6700 Esbjerg

mettelauridsen@gmail.com

Vejledere

Professor Hendrik Vilstrup

Medicinsk Hepato-gastroenterologisk Afdeling V, Århus Sygehus

Nørrebrogade 44, 8000 Århus

Overlæge ph.d. Jeppe Gram

Forskningslektor, Sydvestjysk Sygehus

Finsensgade 35, 6700 Esbjerg

Professor Ove Schaffalitzky de Muckadell

Afdeling for medicinsk mavetarmsygdomme S , Odense Universitetshospital

Sdr. Boulevard 29, 5000 Odense C

Baggrund og formål

Hvad er minimal hepatisk encephalopati?

Minimal hepatisk encephalopati (MHE) er en komplikation, der forekommer hos op til 50% af patienter med levercirrose. MHE er en metabolisk hjernepåvirkning, der patogenetisk hænger sammen med et øget load af cirkulerende neurotoksiske affaldsstoffer, herunder ammonium. Patienter med levercirrose har en katabol metabolisme og dermed øget ammonium load fra muskelnedbrydning. Samtidig bevirker den portale hypertension øget tendens til bakteriel translokation fra tarmen og dermed yderligere øget mængden at blodbårne affaldsstoffer . Under normale forhold omdanner leveren 2 ammoniummolekyler til urinstof, som udskilles via nyrerne . Når leveren svigter ophobes ammonium og sænker krebs cyklus ved at depletere denne for alfa-ketogluterat. Alfa ketogluterat medvirker til at skaffe ammonium af vejen da det sammen med NADPH via glutamat dehydrogenase omdannes til glutamat, som er en neurotransmitter. Hos patienterne kan man observere nedsat mental processeringshastighed, dårlig viso-motor koordination og forstyrret opmærksomhedsniveau (Bajaj 2009, Cordoba 2011). MHE medfører forringet livskvalitet, høj risiko for udvikling af manifest HE og derved en ringere prognose (Groeneweg 1998, Groeneweg 2000). Studier har vist at behandling af MHE med laktulose, rifaximin eller forgrenede aminosyrer bedrer livskvaliteten og bedrer den psykometriske performance (Prasad 2007, Sidhu 2011). Der er således tale om en betydende og hyppig sygdomsenhed, som forholdsvis let lader sig behandle. Derfor er screening af leverpatienter for MHE et væsentligt forskningsområde i hepatologien.

Screening for og diagnostik af MHE

På internationalt plan findes der flere forskellige screeningsmetoder (Amodio 1998, Bajaj 2007, Romero-Gomez 2007, Sharma 2007, Sharma 2010, Weissenborn 2001). Tidligere var Portosystemisk Encephalopati test (PSE-testen) den mest anvendte. Men metoden, som er et blyant-papir testbatteri, er tidskrævende og besværlig at tolke, så den anvendtes kun i Danmark og internationalt i begrænset omfang og er derfor aldrig kommet den brede gruppe af levercirrosepatienter til gode. I erkendelsen af dette er der nu flere computerbaserede screeningstests frit tilgængelige

på internettet. Ingen af dem er fuldt validerede. Derfor er PSE-testen internationalt set fortsat betragtet som en slags guldstandard, selvom der ikke er konsensus eller videnskabelig evidens for, at den fortjener denne status. Der findes endnu ingen hjernetest eller blodprøve, der giver diagnosen MHE med sikkerhed. Udfordringen i forskning inden for hepatisk encephalopati er karakteren af den universelle, men fluktuerende hjernepåvirkning og at alle kendte diagnostiske tests er påvirkelige af mange faktorer herunder alder, søvnmangel og medicinforbrug Det drejer sig således om en meget fluktuerende tilstand uden diagnostisk guldstandard.

Kontinuerte reaktionstidsmålinger

Siden midten af 1980'erne har kontinuerte reaktionstids målinger (CRT) vundet indpas og er nu den foretrukne screeningmetode i Danmark. Brugen er baseret på undersøgelser, der viser at stor intra-individuel varians i reaktionstider kan skelne patienter med HE fra patienter med kronisk organisk hjerneskade (Elsass 1981, Elsass 1984, Elsass 1985, Elsass 1986). CRT-metodens store fordele er at den er hurtig at udføre, at princippet i undersøgelsen er nemt at forstå for patient og læge, at tolkningen er simpel og at det ikke kræves at patienten har computerkendskab. Dette gælder ikke for de øvrige 3-4 computerbaserede diagnostiske tests udviklet i udlandet. Testen udføres via en bærbar computer hvorpå EKHO software findes (Bitmatic, Århus, pris 18.000,-). Softwaren genererer i løbet af testen 150 lydstimuli på 90 dB, som patienten hører i lydisolerede hovedtelefoner. Via en trykknap i den dominante hånd skal patienten reagere så hurtigt så muligt straks lyde opfattes. De 150 lydstimuli leveres med varierende intervaller på 2-6 sekunder. Hele testen tager 10 minutter hvortil tid til instruktion skal lægges – dvs. i alt ca. 15 minutter.

Men CRT-metoden mangler, som de øvrige tilgængelige test, at gennemgå en grundigere validering. Vi ved fra de tidligere studier at cirka 50 % af patienter med levercirrose i en tværsnitsundersøgelse har meget stor intra-individuel varians i reaktionstider (Lauridsen 2011). Variansen eller spredningen i reaktionstiderne beskrives ved et index, der udregnes som 50 percentil/(90 percdentil -10 percentil). Et index under 1.9 er betragtes som unormalt og er angivet med en sensitivtet på 93% og en specificitet på 92% at kunne skelne imellem hepatisk encephalopti, non-hepatisk encephalopati og raske kontroller (Elsass 1985). Der er ingen

tillæringseffekt ved gentagede tests og CRT resultatet påvirkes ikke af intelligens og uddannelsesniveau (Renzi 1965). Både køn og alder påvirker reaktionstiden i mindre grad, men den intra-individuelle varians er ikke påvirket heraf. CRT index er derfor et brugbart mål uanset køn og alder (Lauridsen 2012). CRT resultatet er dårligt korreleret til en af de andre computerbaserede diagnostiske tests, nemlig Critical Flicker Frequency (Lauridsen 2011). Der er derimod en god korrelation imellem MELD score (Model for End Stage Liver Disease) og CRT resultatet, bedst for 90 percentilen (dvs de langsomste reaktionstider ud af de 100 målte med CRT). Vi ved også, at der blandt raske kontrolpersoner er der mindst 10 % der har et abnormt CRT resultat.

En god screeningstest for MHE skal: identificere den population, som kan have gavn af behandling, detektere et behandlingsrespons, være kost-effektiv, medfører mindst mulig ulempe for patienten.

Behandling af hepatisk encephalopati:

Standard behandlingen igennem mange år ved åbenlys hepatisk encephalopati er non-absorberbare disaccarider (laktulose). Igennem de seneste år ar flere præparater afprøvet til behandling af HE. Herunder omtales de bedst dokumenterede behandlinger:

Non-absorberbare disaccarider: Laktulose er i mange studier vist at have en effekt på både livskvalitet og psykometrisk preformance ved behandlingen af hepatisk encephalopati (Bajaj 2010, Prasad 2007, Romero-Gomez 2010, Sharma 2012, Thompson 2010). Virkningsmekanismen er formentlig at pH i mavetarm-systemet sænkes hvorved optaget af NH4- reduceres. Desuden induceres diarre hvorved ammonium i tarmen "skylles" ud. Præparatet er billigt og laktulose har ikke alvorlige bivirkninger - diarre og abdominalia er de hyppigste. Den tidligere omtalte metaanalyse oplyser at 54 ud af 344 oplever diarre og 28 ud af 340 oplever mavesmerter (Eltawil 2012). Man har observeret at virkningen er indtrådt så sent som efter 3 måneder (Prasad 2007).

Rifaximin (Xifaxan) er et bredspektret, ikke absorberbart, antibiotikum, der nu er godkendt til behandling af hepatisk encephalopati. Behandling bedre psykomertisk preformance og livskvalitet (Sanyal 2011, Sidhu 2011, Zullo 2011). Rifaximin udrydder NH4-dannende bakterier i tarmen

og mindsker derved ammonium loadet i blodbanen. Bivirkningerne til rifaximin er få. I en nyligt publiceret metaanalyse findes det at diarre optræder hos 17 ud af 350 og mavesmerter hos 16 ud af 345[(Eltawil 2012)]. Både denne og en tidligere metaanalyse viser at rifaximin tolereres bedre en laktulose[(Jiang 2008)]. Effekten er indtrådt efter 8 uger [(Sidhu 2011)].

Forgrenede aminosyrer/Branch chained amino acids (BCAA): Kendes i Danmark som Bramino, der ikke er et lægemiddel men et ernæringstilskud. Det indeholder de forgrenede aminosyrer leucin, isoleucin og valin og forhandles af Toft Care, Danmark. Disse aminosyrer giver, populært sagt, brændstof til Krebs cyklus og modvirker derved effekten af ammoniumophobning. Det er velkendt at langvarig behandling med BCAA bedre livskvaliteten for cirrosepatienter [(Marchesini 2005)]. Et nyere studie har vist at et tilskud af 30 g forgrenede aminosyrer dagligt forbedre den kognitive funktion hos patienter med MHE og en tidligere episode af HE [(Les 2011)].

Ph.d.-studie

Overordnet formål:

Formålet med dette ph.d-projekt er, at undersøge om CRT metoden egner sig som screeningsværktøj for MHE og om metoden kan identificere et behandlingsrespons.

Overordnet gælder det, at oplysninger om deltagere i projektet beskyttes efter lov om behandling af personoplysninger og lov om patienter retsstilling. Projektet er anmeldt til datatilsynet og der er givet forhåndsgodkendelse. Patientdata håndteres med Case Report Files (CRF) hvorfra data overførers til elektronisk database. Dobbelt back-up af data laves løbende på krypteret USB-stick og ekstern harddisk.

Initiativtagere til projektet er Professor Hendrik Vilstrup, Århus Sygehus, Afdeling V, Professor Ove Schaffalitzky de Muckadell, OUH, afdeling S, ph.d. Jeppe Gram, Sydvestjysk Sygehus Esbjerg samt reservelæge Mette Munk Lauridsen.

Ph.d-projetket udgøres af 3 delstudier.

DELSTUDIE 1:

Formål: At fastsætte normalværdier i den danske population for PSE-resultatet samt yderligere efterprøve cut-off værdien for CRT-metoden, som er fastsat af Elsass et al. I 80'erne.

Forskningsspørgsmål:

Hvor er normalområdet for CRT og PSE-test blandt raske danskere? Er den gængse cut-off værdi på 1,9 for CRT-index god nok?

Hvilken betydning har kronisk sygdom (anden end levercirrose) for CRT- og PSE-test resultaterne?

Hypotese: Raske kontrolpersoner har et CRT-index, der signifikant adskiller dem fra patienter med levercirrose.

Ved anden kronisk sygdom end levercirrose findes ikke øget forekomst af ustabile reaktionstider.

Elsass et al har tidligere fastslået at et CRT-index under 1.9 (index: 50 fraktil/(90 fraktil-10 fraktil) er abnormt hos cirrosepatienter. Denne index-værdi kan skelne imellem cirrosepatienter med åbenlys encephalopati og hjerneskadede patienter. Det kunne være værdifuldt at undersøge hvordan en population af normale klarer CRT-metoden. Egne undersøgelse viser at i en normal population har mindst 10-15 % et abnormt CRT resultat (index under 1,9) og det giver anledning til udvidelse at det normal-materiale vi har mhp. om cut-off værdien skal revurderes. Eller om de mange unormale resultater hos normale simpelthen bare skyldes manglende motivation sammenlignet med patienter, der risikere at miste deres kørekort som konsekvens af et abnormt resultat. Mulig confounder kunne også være søvnmangel.

Patienter med anden kronisk sygdom end levercirrose er ikke undersøgt med CRT metoden. Dog fremgår det af et arbejde af Elsass et al. (Elsass 1986), at patienter med kronisk nyresvigt/dialyse-patienter ikke har et abnormt CRT index – altså stor variation i

reaktionstider. Dette er påfaldende da netop kronisk nyresvigt med uræmi kan forårsage metabolisk encephalopati. Tanken bag at undersøge kronisk syge patienter er at afgøre om/udelukke at, hvilken som helst kronisk lidelse kan forårsage ustabilitet i reaktionstider.

Metode: Tværsnitsstudie, hvor 100 helt raske personer testes med både CRT- og PSE-metoden mhp at fastslå normalområder i den danske befolkning og yderligere efterprøve om den gængse CRT-index cut off værdi på 1.9 er hensigtsmæsssig. De to tests forgår samme dag.

50-60 patienter i ambulante forløb med andre kroniske lidelser end levercirrose testes med både CRT- og PSE-metoden. Desuden laves en komorbiditetsscore mhp at afklare hvordan kronisk sygdom influere på stabiliteten i reaktionstiderne. Der planlægges at undersøge 15 patienter med hjertesvigt, 15 med kronisk obstruktiv lungesygdom, 15 patienter med reumatoid artritis og 15 patienter med diabetes. Der ligger ikke en styrkeberegning bag valget af populationsstørrelsen. Der er som nævnt ikke dansk normalområde til PSE testen.

Continous Reaction Times (CRT)
Se beskrivelse af testen under introduktionen. Udførelse af testen holdes på så få hænder som muligt og undersøgelsen finder sted i et uforstyrret lokale i dagtid (kl 8-16) for at opnå så standardiserede forhold som muligt. Varighed 15 min. for 150 målinger.

Portosystemisk Encephalopati- test (PSE-test)

Papir-blyant testbatteri. Varighed ca. 20-30 min. Test manual er oversat til dansk af projektansvarlige med tilladelse fra det internationale selskab for hepatisk encephalopati og nitrogen metabolisme (ISHEN), der er rettighedsindehaver. Testen består af 5 sub tests. Især tolkningen af linetesten kan volde problemer. Forsøgsansvarlige er oplært af udvikleren af PSE-testen Professor K. Weissenborn fra Medizinsche Hochschule Hannover for at undgå systematisk forkert brug af testen. Resultatet af PSE-testen er den psykometriske hepatiske encephalopati score (PHES). Forsøgsansvarlige samt et par oplærte forskningsmedhjælpere varetager undersøgelsen i forbindelse med ph.d.-projektet.

Charlson Score: Charlson scoren eller Charlson indexet er et komorbiditets index der forudsiger 10-års mortaliteten hos patienter med flere konkurrende lidelser. Hver kronisk lidelse registreres og gives en point-værdi afhængig af hvor dødelig sygdommen er. Patienten

udfylder selv eller med hjælp et spørgeskema, hvor der spørges til 19 sygdomskategorier. Jo højere Charlson index desto ringere prognose.

Blodprøver: Alle kronisk syge kontroller får, efter samtykke målt, leverenzymer på alm. blodprøve i forbindelse med at der alligevel tages blodprøver til deres vanlige ambulante opfølgning.

Statistik: Resultater fra CRT metoden og PSE benyttes til at beregne sensitivitet og specificitet samt PPV og NPV idet guldstandard for diagnostik af MHE antages at være PSE. Evt. kovariater af interesse (f.eks. køn, alder, søvnmangel, andre diagnoser) kan evt. inddrages ved en regression og de beregnede mål (sensitivitet, specificitet, PPV og NPV) vil da blive baseret på regressions modellen. I forvejen ved vi at alder og køn ikke påvirker CRT indexet.

Styrkeberegning:

Udfaldsrummet på CRT index hos kontrolpersoner er efter egen erfaringer 1,7-3,5. Variansen er ((3,5-1,7)x0,95)/4=0,43.

Vi kan udregne, på basis af ca. 92 målinger af CRT foretaget tidligere på raske kontroller, at interperson middelværdi og spredning på CRT index er 2.56 ± 0.64 (SD).

Formålet er at påvise en korrelation imellem CRT index og PSE-resultatet. Da der ikke er noget dansk normalmateriale på PSE testen og dette ønskes inkluderes efter skøn 100 kontrolpersoner og 50-60 kronisk syge.

Studiepopoulation: Raske kontrolpersoner, i alt 100 med alder over 18 år. Vi vil godt opnå inklusion af en del midaldrende personer mhp at matche denne raske gruppe til patientgruppen, hvor de fleste forventes at være imellem 40-70 år. Gruppen af personer udvælges også til en vis grad i forhold til job og uddannelsesniveau.

50-60 personer med kroniske lidelser andre end levercirrose. F.eks. KOL, diabetes, hjertesvigt, reumatoid artritis. Patienterne skal have stabile forløb på testtidspunktet og følges i ambulant regi.

Rekruttering: Der laves opslag med information om muligheden for at blive kontrolperson i studiet. Opslagene hænges op i de medicinske ambulatorier på Sydvestjysk Sygehus samt på 2

arbejdspladser efter aftale med lederen på stedet. Folk kontakter selv forsøgsansvarlige via telefon hvis de har lyst til at deltage. Hvis de har mod på at deltage mødes forsøgsansvarlige med dem hvor der gives grundig information om projektet. Der udleveres skriftligt materiale, herunder "Før du beslutter dig". Den potentielle deltager gives tid til at læse materialet alene. Herefter afklares eventuelle spørgsmål og samtykkeerklæringen (S2) underskrives såfremt personen fortsat ønsker at deltage. Der ydes ikke vederlag for deltagelse.

Inklusionskriterier:

1. Alder >18
2. Skriftligt informeret samtykke
3. Taler og forstår dansk

Eksklusionskriterier:

1. Indtag af psykoaktive stoffer inden for 6 dage af testen
2. Organisk hjernesygdom (f.eks. tidl. apoplexi, demens)
3. Hypothyroidisme
4. Nyresvigt (creatinin> 150 mmol/L)
5. Sepsis eller blødning inden for en uge forud for tests
6. Alvorlige søvnforstyrrelser
7. Indtag af alkohol på testdagen

Videnskabsetisk redegørelse:

Der er ingen ulemper for de raske personer ved at gennem gå CRT og PSE-test bortset fra at det kræver noget tid. Ca. 30 min i alt. Der er ingen gevinst for deltageren. Der ydes intet vederlag for deltagelse i projektet. Men der vil ydes transport godtgørelse i det omfang det er nødvendigt. Alle deltagere modtager information om projektet skriftligt og mundtligt jvf. afsnittet " Patientinformation og publikation". Det skønnes at være etisk korrekt af bede raske personer om at bruge tid på deltagelse da vi herved opnår ny viden mht. behandling af tidlige stadier af encephalopati.

Økonomi:

Se det samlede drifts budget under afsnittet ” Tidsplan og overordnet budget”. Skøn over delprojeket 1’s omkostninger følger herunder:

Aflønning af forskningsassistent som hjælper med udførelse af tests i Odense og Esbjerg	100.000 kroner anslået	Region Syddanmarks regionale forskningspulje ansøget marts 2013
Ialt	100.000 kroner	

Finansiering sker via private fonde samt Region Syddanmarks Regionale forskningspulje som er ansøgt om penge til løn til forskningsassistenter både i 2012 og 2013.

DELSTUDIE 2:

Formål: At undersøge om CRT metoden og PSE-metoden er enige om, hvilke patienter med levercirrose, der har MHE samt hvordan de psykometriske testresultater korrelerer med patienters livskvalitet. Vi vil også undersøge hvilken test (CRT eller PSE) der korrelere bedst Child Pugh og MELD score.

Hypoteser:

1. CRT resultatet er korreleret til PSE resultatet og de kliniske scores MELD og Child-Pugh.
2. Livskvalitetsscores er ringere hos patienter med lavt CRT index (og abnormt PSE testresultat).

Metode: Tværsnitsundersøgelse af patienter med kendt levercirrose.

Ved inklusionen af levercirrosepatienter foretages følgende tests:

1) CRT test
2) Livskvalitets scores (SIP og SF-36)
3) Hvis patienterne er samlevende testes samlever/ægtefælle efter samtykke med IQCODE som er et spørgeskema, der bruges til at afdække hvordan nærmeste pårørende opfatter patientens sygdom.
4) PSE test
5) MMSE test som laves for at sikre ensartet udspørgen til orientering i tid, sted og egne data samt screene for hukommelsesforstyrrelser.
6) Blodprøver: Hæmoglobin (1 ml EDTA-blod), blodsukker (1 ml, citrat-flourid.EDTA-blod, pink glas), albumin, bilirubin, ALAT, BASP, creatinin, natrium, CRP, kalium, TSH, Magnesium (1 ml li-heparin plasma, grønt glas), KFNT (1ml Na-citrat plasma, blåt glas), venøs ammonium (1 ml EDTA-plasma, lilla glas) desuden vil en blodprøve udtaget ved inklusion i delstudie 2 blive gemt i biobank med henblik på senere analyse. For diabetes patienter måles tillige HbA1c. Der udtages i alt 5 prøveglas med blod

svarende til højst 25 ml blod. Blodprøverne analyseres på klinisk biokemisk afdeling på Sydvestjysk Sygehus Esbjerg for de patienter der inkluderes her og på kliniks biokemisk afdeling på OUH for de patienter der inkluderes her. Biobanken vil fysisk befinde sig på Sydvestjysk Sygehus. Forsøgsansvarlige varetager transport af blodprøver fra OUH til biobank i Esbjerg. Blodprøverne vil blive opbevaret i personhenførbar form med påtegnet deltagernummer.

7) Højde og vægt måles mhp at opnå et mål for patientens ernæringsstatus.
8) Charlson Komorbiditetsscore udregnes.
9) Evt. DEXA-scanning som mål for muskelmasse

Der skal desuden registreres information om

1) Medicin forbrug aktuelt og igennem de sidste 3 måneder. Særligt kan brug af antibiotika, sedativa og opioider har interesse.
2) Alkohol anamnese. Nuværende forbrug samt tidligere forbrug ”kasse-år”.
3) Uddannelsesniveau. Antal år i folkeskole og antal år på videregående uddannelse. Aktuelt job.
4) Charlson Score som mål for komorbiditet som diabetes, hjertesygdom, neurologiske lidelser, lungesygdom.
5) Årsag til levercirrose.
6) Objektiv vurdering: Er patienten cerebralt normal. Ascites.

For nærmere beskrivelse af CRT og PSE se baggrundsafsnit samt under delstudie 1.

Livskvalitetsscore

Sickness Impact Profle (SIP) spørgeskemaet er i et studie om MHE vist at kunne detektere et behandlingssrespons. [Prasad 2007]. SIP danner et mål for opfattet helbredsstatus, som er følsomt nok til at opfange ændringer eller forskelle i helbredsstatus som indtræffer over tid eller mellem grupper. SIP er et funktionelt statusinstrument, der består af 136 udsagn, som beskriver sygdoms-relaterede adfærds dysfunktioner. Disse udsagn kan inddeles i 12

subskalaer. Tre subskalaer vedrørende fysisk funktion og fire om psykosocial funktion desuden spørgsmål vedr. arbejdsevne, søvn, fødeindtag mm. Håndteringstiden er angivet til at ligge mellem 20 – 30 min. ved interviewhåndtering (Bergner 1981). Hvis der er den mindste tvivl om hvorvidt patienten er i stand til at læse og forstå de mange spørgsmål, der afdækker livskvalitet inden for 12 områder, skal patienten guides igennem udfyldelsen af skemaet. Forsøgsansvarlige samt et oplærte forskningsmedhjælpere varetager undersøgelsen.

SF-36: Et spørgeskema patienterne selv kan udfylde eller som kan udfyldes via en interviewer. Skemaet indeholder 36 punkter, der afdækker selvvurderet helbred. SF-36 består af 36 items, der er inddelt i otte separate multiitemskalaer og en enkelt 'Ændringer i alment helbred (HT)' spørgsmål. De 8 emner drejer sig om fysisk funktion, begrænsninger – fysisk betinget, fysisk smerte, alment helbred, energi, social funktion, begrænsninger – psykisk betinget, psykisk velbefindende. Alle skalaerne scores, så en høj score indikerer en positiv helbredsstatus. Råscorerne i hvert domæne transformeres til en score, som repræsenterer respondentens relative position på et kontinuum mellem 0 og 100 %. Håndteringstid ca. 20 min.

MMSE

MMSE indeholder en række korte, simple opgaver til belysning af otte forskellige typer kognitive funktioner. Præstationerne scores og summeres i en råscore der går fra 0 - 30, hvor 30 udgør den 'bedste'(fejlfri) præstation. MMSE er oprindeligt udviklet med henblik på at måle kognitive færdigheder i bred forstand hos ældre samt at registrere eventuelle ændringer i det kognitive funktionsniveau over tid, men MMSE er ikke specielt konstrueret til at identificere tegn på demens. Testen medtages i dette studie for at teste pateintens orientering i tid og sted og for at screene for hukommelsesproblemer. Håndteringstid ca 10 min.

IQCODE: Informant Questionaire on Cognitive Decline in the Elderly er udviklet til brug ved demenstilstande og andre lidelser hvor patienten har ringe sygdomsindsigt og hvor det har værdi at måle sygdomsudviklingen via nærmest pårørende. Spørgeskemaet består af en række af 16 spørgsmål vedrørende patientens hukommelse, apraksi, indlæringsevne, beslutningsdygtighed og regnefærdigheder. Svarene omsættes til pointværdier og en total score udregnes (Jorm 2004).

Biobank

Kliniske oplysninger og biologisk materiale (blodprøve, 4 prøveglas, 5 ml) ønsker vi at opbevare i biobank efter foreliggende retningslinjer fra Datatilsynet. Formålet med biobanken er at sikre det biologiske materiale til fremtidige studier. Opbevaring af materialet sker i kodet form med påtegnet deltagernummer der vil være personhenførbar. Blodprøverne vil blive frosset ned og gemt til senere analyser. Der oprettes således en biobank i en 7 års periode efter forsøgets afslutning. Materialet destrueres herefter. Ved anvendelse af materialet fra biobanken søges ny tilladelse fra etisk komite. Det er kun en blodprøve udtaget ved inklusion i delstudie 2 der opbevares i biobank.

Statistik og effektmål:

Primære effektmål i tværsnitsstudiet er korrelationen mellem PES og CRT. Sekundære effektmål er korrelationen mellem livskvalitet, MELD, Child Pugh score og CRT.

Styrkeberegning:

Ikke findes ingen danske normalværdier og spredning på PSE testen. Derfor kan en sikker styrkeberegning ikke laves.

Ved egne tidligere studier på området er spredning og middelværdi blandt cirrose patienter for CRT testen fundet på basis af 180 målinger: Interperson middelværdi og spredning på CRT index i cirrosepatienter: 2.00 ± 0.80 (SD), Varians 0.64.

Der inkluderes efter skøn ca. 100 personer.

Studiepopulation: Studiepopulationen skal udgøres af patienter med levercirrose enten biopsiverificeret eller konstateret ved billeddiagnostik (UL eller CT scanning) og passende klinik (cirrose stigmatiseret ved objektiv undersøgelse og påvirket lever syntesefunktion vurderet biokemisk ved nedsat KFNT og albumin, passende anamnese). Patienterne må ikke have åbenlys HE dvs. de skal være West Haven klasse 0 eller 1.

Rekruttering: Patienterne rekrutteres fra Sydvestjysk Sygehus Esbjerg, Odense Universitets Hospitals afdeling S samt Sydvestjysk Sygehus Esbjerg. Identificering af egnede patienter skal

ske ved hjælp af faste læger på de nævnte matrikler samt ved hjælp af en yngre læge, der tilknyttes projektet. Hvis der kan findes økonomiske midler til det, tilknyttes ligeledes en projektsygeplejerske 7 1/2 timer om ugen på afdeling S, OUH og medicinsk gastroenterologisk afdeling på Sydvestjysk Sygehus. Patienterne kan enten selv kontakte de forsøgsansvarlige som reaktion på rekrutterings-opslag, der ophænges i involverede afdelinger. Eller de adspørges af fast læge under almindelig ambulant kontrol om de forsøgsansvarlige må kontakte dem angående mulig deltagelse i projektet. Der vil til interesserede patienter udleveres skriftligt materiale som der gives god tid til at gennemlæse. Når det passer patienten evt. ved næste ambulante kontrol afklares eventuelle spørgsmål. Hvis patienten ønsker at deltage underskrives samtykkeerklæring (S4).

Inklusionskriterier:

1) Alder >18
2) Levercirrose verificeret ved biopsi eller passende klinik og biokemi samt billeddiagnostik.
3) Skriftligt informeret samtykke
4) Taler og forstår dansk

Eksklusionskriterier:

1. Indtag af psykoaktive stoffer inden for 6 dage af testen
2. Organisk hjernesygdom (f.eks. tidl. apoplexi, demens)
3. Hypothyroidisme
4. Nyresvigt (creatinin> 150 mmol/L)
5. Hyponatriæmi (Na < 125 mmol/L)
6. Sepsis eller blødning inden for en uge forud for tests.
7. Alvorlige søvnforstyrrelser
8. Aktuel behandling med laktulose, rifaximin eller BCAA
9. Indtag af alkohol på testdagen

Videnskabsetisk redegørelse: Det er forbundet med ulempe at deltage i projektet da det kræver, at patienten bruger ekstra tid under ambulante besøg. Desuden kan det være forbundet med ulempe at skulle have taget blodprøver, men det er en almindelig del af de ambulante kontroller. Udgifter til transport dækkes via patientbefordringen på vanlig vis. Der ydes ikke vederlag for deltagelse i forsøget, men der vil være mulighed for at få lidt at spise under vejs da udførelsen af alle de nævnte tests forventes at tage op mod 1-2 timer. Den grundige evaluering patienten gennemgår forventes at komme patienten til gavn da eventuelle problemer med den cerebrale funktion opdages. Deltagelse i projektet vil bidrage med ny viden inden for området og på sigt kan denne viden ligge til grund for at fokus på behandling af tidlige stadier af hepatisk encephalopati skærpes. Patienterne modtager grundig information om, hvad deltagelse indebærer og tilbydes at fortsætte ind i den longitudinelle del af projektet. Alle deltagere modtager information om projektet skriftligt og mundtligt jvf. afsnittet " Patientinformation og publikation". Patienterne vil modtage grundig information om biobanken og give samtykke til at blod må indgå heri på særskilt samtykke erklæring.

Økonomi:

Se det samlede drifts budget under afsnittet " Tidsplan og overordnet budget". Skøn over delprojeket 1's omkostninger følger herunder:

Blodprøver og DEXA sacnning		Udgifter dækkes af de involverede afdelinger
Aflønning af forskningsassistent som hjælper med udførelse af tests i Odense og Esbjerg (7 ½ timer per uge hvert sted)	100.000	Region Syddanmarks forskningspulje ansøges samt der er opnået støtte med 266.000 i alt til TAP løn fra OUHs frie forskningsmidler
Tryksager, forplejning og diverse	12.000	Dækket af Karola Jørgensens Fond samt involverede afdelinger
PC til artikelskrivning og	25.000	Dækkes af OUHs frie

databehandling andet apparatur		forskningsmidler
Udenlandsophold mhp oplæring i brug af PSE test og ophold på udenlandsk forskningsinstitut (3 mdr.)	76.000	Dækkes af Karola Jørgesens Fond samt Vs forskningsmidler og rejselegater
Ialt	213.000	

Finansiering sker via private fonde samt Region Syddanmarks Regionale forskningspulje som er ansøgt om penge til løn til forskningsassistent. De involverede afdelinger dækker udgifter til blodprøver da disse ikke adskiller sig væsentligt fra de prøver der tages rutinemæssigt. Rejseudgifter dækkes til dels af afdeling V Århus Sygehus samt via rejselegater. Der planlægges længere ophold i udenlandsk forskningslaboratorium forhåbentligt Barcelona, Spanien.

DELSTUDIE 3:

Formål: At undersøge om CRT-metoden er i stand til at detektere ændringer i den cerebrale funktion under behandling for encephalopati hos patienter med levercirrose (men uden klinisk erkendelig encephalopati).

Hypoteser:

1) CRT-metoden kan detektere ændringer i patienternes cerebrale funktion.
2) CRT, PSE og livskvalitetsscores ændres til det bedre efter påbegyndt behandling og de patienter, der opnår det laveste CRT-index har størst gavn af laktulose, BCAA og rifaximin sammenlignet med patienter med højt CRT-index.

Metode: Prospektivt, blindet, randomiseret interventionsstudie af "anti-encephalopatibehandling" af patienter med mulig MHE:

Alle inkluderede cirrosepatienter indgår efter samtykke i en balanceret randomisering efter stratifikation (på basis af CRT over eller under 1.9) til:

1) behandling med både rifaximin, laktulose og forgrenede aminosyrer (Bramino) som er den bedst dokumenterede behandling til patienter med manifest hepatisk encefalopati.

2) Placebo laktulose, rifaximin og forgrenede aminosyrer.

Både anti-encephalopati-gruppen og placebo-gruppen opstarter behandling ved inklusion i delstudie 2. Alle de inkluderede patienter testes igen efter 3 måneder.

Ved opfølgning efter 3 måneder foretages der altså følgende tests:

1) CRT test
2) Livskvalitets score (SIP og SF-36)
3) PSE test
4) MMSE test
5) Blodprøver: Hæmoglobin (1 ml EDTA-blod), blodsukker (1 ml, citrat-flourid.EDTA-blod, pink glas), albumin, bilirubin, ALAT, BASP, creatinin, natrium, CRP, kalium, TSH, Magnesium (1 ml li-heparin plasma, grønt glas), KFNT (1ml Na-citrat plasma, blåt glas), venøs ammonium (1 ml EDTA-plasma, lilla glas).
6) Højde og vægt måles
7) Evt. ny DEXA-scanning

Der skal desuden registreres igen information om

7) Medicin forbrug aktuelt
8) Alkohol anamnese. Nuværende forbrug
9) Objektiv vurdering: Er patienten cerebralt normal? Ascites?

Forsøgsmedicin

Patienterne randomisers til behandling med standard behandlingen for hepatisk encephalopati eller placebomedicin. Medicinen udleveres gratis til patienterne. I interventionsstudiet gives rifaximin, laktulose og forgrenede aminosyrer i kombination. Alle 3 stoffer gives samtidig i en follow up periode på 3 måneder.

Placebomedicin

Den gruppe af patienter, der randomiseres til behandling med placebo medicin tilbydes både placebo rifaximin, laktulose og Bramino. Placebo-rifaximin fremstilles fra producenten af rifaximin. Laktulose vil blive svær at efterligne nøjagtigt. Vi planlægger i første omgang at afprøve 20% glukose tilsat fortykningsmiddel (f.eks Attylet, Toft Care). Der er, så vidt vi ved, ikke lavet en gængs brugt placebo til laktulose. De forgrenede aminosyrer er på pulverform og placebo til dette bliver L-alanin i pulverform.

Statistik og effektmål:

Primære effektmål i interventions studiet er forskellen i CRT mellem ”anti-encephalopati”-behandlingsgruppen og placebogruppen. Sekundære effektmål er reduktion i antal komplikationer (indlæggelser, HE episoder og død) bedring af livskvalitet og bedring af PSE samt reduktion i arteriel ammonium.

Analysen af de longitudinelle data fremkommet ved follow-up studiet vil blive analyseret ved en passende hierarkisk model idet der tages hensyn til de forskellige bidrag til den tilfældige variation (indenfor patient og tid, mellem followup tider og mellem patienter). Dette er essentielt i forhold til en korrekt estimering af variansen og dermed bestemmelse af en evt. behandlings effekt. Denne del af analysen vil også undersøge eventuelle divergerende behandlings effekter mellem grupper inkluderet på forskellige diagnose grundlag. Dette gøres ved passende interaktions led i den longitudinelle regressions analyse

Styrkeberegning:

Middelværdi og spredning på forskellen mellem 2 CRT målinger hos den samme cirrosepatient er 0,28 ± 0,35.

Der skal med de anførte type 1 og type 2 fejl (se delstudie 2) randomiseres 32 personer med CRT-index under 2,5. Idet der forventes drop-outs er målet at randomisere 44 personer med CRT index under 2,5.

Af de personer, der indgår i tværsnitsstudiet (delprojekt 2) forventes 75% at have index under 2,5 og disse kan tilbydes at indgå i den balancerede randomisering.

Alle de der testes til tværsnitsstudiet tilbydes at fortsætte som deltagere i follow up studiet.

Population: Studiepopulationen skal udgøres af patienter med levercirrose enten biopsiverificeret eller konstateret ved billeddiagnostik (UL eller CT scanning) og passende klinik (cirrose stigmatiseret ved objektiv undersøgelse og påvirket lever syntesefunktion vurderet biokemisk ved nedsat KFNT og albumin, passende anamnese).

Rekruttering: Som ved delprojekt 2.

Inklusionskriterier: Som for delstudie 2.

Eksklusionskriterier: Som for delstudie 2. Desuden:

Patienter med tidligere episoder af hepatisk encephalopati
Patienter med en eller flere tidligere episoder af HE kan som udgangspunkt inkluderes i studiepopulationen, hvis de ikke allerede er i behandling med både rifaximi, laktulose og BCAA eller har åbenlys HE. I de efterfølgende statistiske analyser kan de tidligere encephalopate eventuelt indgå i en særskilt analyse. Men det vil under alle omstændigheder være værdifuldt at vide om CRT kan detektere bedring eller tilbagefald i denne gruppe af patienter også.

Udvikling af hepatisk encephalopait under follow up
I den behandlede gruppe: Hvis en patient fra gruppen af patienter under behandling med både rifaximin, laktulose og BCAA under follow up perioden udvikler åbenlys HE behandles udløsende faktor efter vanlige retningslinier og patienten udgår.
I den placebo-behandlede gruppe: Udløsende faktor behandles efter vanlige retningslinier og patienten udgår.

Videnskabsetisk redegørelse:
Patienternes ulempe ved deltagelse består i den gene, der er ved blodprøvetagningen, tidsforbruget (op med 2 timer ved hver follow up) samt i de mulige bivirkninger der er til forsøgsmedicinen. Resultaternes gavn for vores viden om hepatisk encephalopati skønnes at overstige den ulempe patienterne udsættes for. Deltagelse i projektet kan komme den enkelte projektdeltager til gode idet det forventes at nogle af de behandlede patienter opnår bedring i

livskvalitet. Patienterne ydes intet vederlag for deres deltagelse i projektet. Der vil yders befordringsgodtgørelse som ved enhver anden ambulant opfølgning. Deltagelse i projektets behandlings-del er forbundet med risiko for bivirkninger. Men kun de patienter, som vurderes at have en vis grad af minimal hepatisk encephalopati tilbydes behandling og i disse tilfælde skønnes det at effekten af behandlingen overskygger ulempen fra beskedne bivirkninger. Ved betydende bivirkninger vil behandlingen naturligvis stoppes eller nedgraderes til opfatte færre præparater.

Lægemiddelforsøg eller ej

Rifaximin er et lægemiddel godkendt til behandling af hepatisk encephalopati og fungere kun som værktøj i dette projekt. Bramino er et ernæringsprodukt og ikke et lægemiddel. I dette forsøg betragtes det som et kosttilskud og er som Rifaximin et værktøj som skal hjælpe os med at inducere en målbart ændring i de psykometriske tests. Laktulose er et håndkøbslægemiddel og standard behandling ved hepatisk encephalopati på alle landets gastroenterologiske afdelinger. De 3 medikamenter fungere altså alle som værktøj i dette projekt mhp at inducere at målbart behandlingsrespons.

Etiske overvejelser i forbindelse med randomisering.

Under randomiseringen vil nogle patienter tilbydes behandling og andre ikke. Screening for og behandling af minimal hepatisk encephlopati er ikke noget man rutinemæssigt gør i Danmark og der er endnu ikke nationale guidelines, der tilsiger at man bør gøre dette. De der ikke tilbydes behandling får derfor det der er ”standard of care” de fleste steder i landet og det skønnes derfor at være etisk forsvarligt at have en placebo-gruppe med i forsøget. Skulle en patient som indgår i studiet udvikle åbenlys HE tilbydes vedkommende den gængse behandling herfor.

Økonomi:

Forskningssygeplejerske:		
Esbjerg	260.000	Region Syddanmarks regionale

Odense	260.000	forskningspulje er ansøgt samt i alt 266.000 kroner er modtaget til TAP løn fra OUHs frie forskningsmidler.
Forsøgsmedicin		
- rifaximin	165.600	Dækkes af producenten
- laktulose	21.000	Betales af de involverede afdelinger
- Bramino	17.000	Betales af OUHs frie forskningsmidler
Placebomedicin	100.000	Placebo Rifaximin betales af producenten af Rifaximin. Udgift til udvikling af øvrig placebomedicin og udgifter til sygehusapotek søges dækket via private fonde herunder Lundbeck Fonden.
I alt anslået	823.600,-	

Medicinen er under alle forhold gratis for forsøgsdeltageren og udleveres efter randomiseringen og ved follow up-besøgene.

Rifaximin: Producenten sponsorerer medicin til projektet. I alt skal der bruges tabletter til behandling af 22 personer i 3 måneder. Døgndosis er 1100 mg fordelt på 2 doser a 550 mg (2 tabletter/døgn). Samlede omkostninger (90 døgn x 2 tabl x 23 personer = 4.140 tabletter x ca 40 kroner/stk) ialt 165.600 kroner. Producenten leverer placebo Rifaximin tabletter.

Bramino gives i dosis 17 g dagligt. Prisen for Bramino er 730 kroner for 500 g. Pris for behandling af 22 patienter i 3 måneder: Ca 50.000,-. Denne pris er der give et væsentligt afslag på. Den endelige pris bliver ca. 17.000,-

Laktulose behandlingen hører ind under almindelig behandling af mange cirrosepatienter, så det betales via de pågældende afdelinger. Dosis for laktulose under forsøget er 25 mL x 3 dagligt (i alt til 23 patienter i 3 måneder: 155.250 mL=155 L a 135 kr/L=ca. 21.000 kr).

Tidsplan

Studiet påbegyndes 1. November 2012 og slutter 31. oktober 2015.

1. november 2012 - 31.oktober 2013: 1 uges besøg på Medical School Hannover ved Dr.med Karin Weissenborn, med henblik på oplæring i PSE. Inkludering af patienter og afvikling af biostatistik kursus i foråret 2013. Opbygning af referencer. Artikel til Ugeskrrift for Læger (og evt. Sygeplejersken) med oversigt over sygdomsenheden: Minimal hepatisk encephalopati.

1. november 2013 - 30. april 2014: Fortsat inklusion af patienter. Artikel baseret på tværsnitsdata skrives. 3 måneders follow-up.

1. maj 2014 – 31. juli 2014: Besøg på Hospital Vall Hebron i Barcelona hos Dr.med Juan Cordoba mhp. samarbejde om livskvalitetsmåling.

1. august 2014 – 30. januar 2015: Artikel om follow-up resultater/behandlingsresultater skrives.

1. februar 2015 – 31. oktober 2015: Sammenskrivning af projektet og afvikling af de sidste kurser

Budget

Løn til læge Mette Munk Lauridsen dækkes via 1 års fakultetsstipendium fra Syddansk Universitet, Institut for Regional Sundhedsforskning (forskningsrådet ved Sydvestjysk Sygehus) har givet 1 års løn og desuden stillet garanti for yderligere et års løn såfremt Region Syddanmark ikke imødekommer ansøgning om løn. Region Syddanmarks ph-d-pulje ansøges om 1 årsværk. Ansøgningsfrist er til marts 2013.

Patientinformation og publikation

Ved ambulante kontakter angående udredning og behandling af levercirrose kontaktes patienten og informeres om muligheden for at deltage i et videnskabeligt forskningsprojekt. Foruden skriftligt informationsmateriale om projektet udleveres folderen ”Dine rettigheder som forsøgsperson i et biomedicinsk forskningsprojekt”. Patienten informeres om mulighed for at han/hun kan have en bisidder med til et informationsmøde og at der er mulighed for betænkningstid inden eventuelt samtykke. Giver patienten en positiv tilbagemelding aftales tid til yderligere information.

Ved mødet gentages den mundtlige information af den projektansvarlige læge eller af dennes stedfortræder, som også har tilknytning til projektet. Mødet vil finde sted i et uforstyrret lokale, egnet til formålet, og den ansvarlige er opmærksom på tilstrækkelig tid, så skriftlig materiale om nødvendigt kan genlæses og eventuelle spørgsmål besvares. Der informeres om omfanget af projektet, ulemper og mulige bivirkninger og om, at patienten ikke nødvendigvis selv vil have direkte udbytte af deltagelse, men at vi håber, at resultaterne kan bidrage til ny behandling af andre med samme sygdom. I den forbindelse er den ansvarlige opmærksom på, at patienten kan have frasagt sig viden om egen helbredstilstand. Efter betænkningstid kan patienten give informeret samtykke (der anvendes Videnskabsetisk komité fortrykte formular S2 og S4), idet han/hun skriver under på at have modtaget både skriftlig og mundtlig information. I forbindelse hermed tager patienten også stilling til eventuelt bidrag til donation af biologisk materiale til en biobank.

Projektet kan påbegyndes umiddelbart efter informeret samtykke er givet. Opstår der tvivl eller spørgsmål kan patienten til enhver tid kontakte projektlederen, der også vil være kontaktperson.

Studiet udføres i henhold til Helsinki II deklarationen og vil først blive sat i gang efter godkendelse fra den Videnskabsetiske Komité for Region Syddanmark. Studiet er registreret i databasen Clinicaltrials.gov.

Projektets resultater søges offentliggjort i internationalt Hepatologisk/neurologisk fagtidsskrift. Såvel positive som negative forsøgsresultater vil blive publiceret. Aftale om forfatterskaber følger Vancouver-deklarationen.

Udelukkelse / afbrydelse af forsøget

Afbrydelse af forsøget kan ske hvis patienten ønsker det f.eks. som følge af medicinbivirkninger.

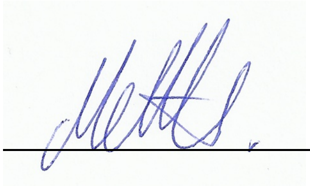

Mette Munk Lauridsen

Ph.d.-studerende

Referencer

Amodio P, Marchetti P, Del Piccolo F, et al. (1998) Study on the sternberg paradigm in cirrhotic patients without overt hepatic encephalopathy. Metab Brain Dis.13:159-172.

Bajaj JS, Saeian K, Verber MD, et al. (2007) Inhibitory control test is a simple method to diagnose minimal hepatic encephalopathy and predict development of overt hepatic encephalopathy. Am J Gastroenterol.102:754-760.
AJG1048 [pii]
10.1111/j.1572-0241.2007.01048.x

Bajaj JS, Saeian K, Schubert CM, et al. (2009) Minimal hepatic encephalopathy is associated with motor vehicle crashes: The reality beyond the driving test. Hepatology.50:1175-1183.
10.1002/hep.23128

Bajaj JS (2010) Review article: The modern management of hepatic encephalopathy. Aliment Pharmacol Ther.31:537-547.
APT4211 [pii]
10.1111/j.1365-2036.2009.04211.x

Cordoba J (2011) New assessment of hepatic encephalopathy. J Hepatol.54:1030-1040.
S0168-8278(10)01102-5 [pii]
10.1016/j.jhep.2010.11.015

Elsass P, Christensen SE, Ranek L, Theilgaard A, Tygstrup N (1981) Continuous reaction time in patients with hepatic encephalopathy. A quantitative measure of changes in consciousness. Scand J Gastroenterol.16:441-447.

Elsass P (1984) Number connection test and continuous reaction times in assesment of organic and metabolic encephalopathy: A comparative study. Acta pharmacol et toxicol.54:115-119.

Elsass P, Christensen SE, Mortensen EL, Vilstrup H (1985) Discrimination between organic and hepatic encephalopathy by means of continuous reaction times. Liver.5:29-34.

Elsass P (1986) Continuous reaction times in cerebral dysfunction. Acta Neurologica Scandinavica.73:225-246.

Eltawil KM, Laryea M, Peltekian K, Molinari M (2012) Rifaximin vs conventional oral therapy for hepatic encephalopathy: A meta-analysis. World J Gastroenterol.18:767-777.
10.3748/wjg.v18.i8.767

Groeneweg M, Quero JC, De Bruijn I, et al. (1998) Subclinical hepatic encephalopathy impairs daily functioning. Hepatology.28:45-49.
S0270913998002699 [pii]
10.1002/hep.510280108

Groeneweg M, Moerland W, Quero JC, Hop WC, Krabbe PF, Schalm SW (2000) Screening of subclinical hepatic encephalopathy. J Hepatol.32:748-753.
S0168-8278(00)80243-3 [pii]

Jiang Q, Jiang XH, Zheng MH, Jiang LM, Chen YP, Wang L (2008) Rifaximin versus nonabsorbable disaccharides in the management of hepatic encephalopathy: A meta-analysis. Eur J Gastroenterol Hepatol.20:1064-1070.
10.1097/MEG.0b013e328302f470
00042737-200811000-00003 [pii]
Jorm AF (2004) The informant questionnaire on cognitive decline in the elderly (iqcode): A review. Int Psychogeriatr.16:275-293.

Lauridsen MM, Jepsen P, Vilstrup H (2011) Critical flicker frequency and continuous reaction times for the diagnosis of minimal hepatic encephalopathy: A comparative study of 154 patients with liver disease. Metab Brain Dis.26:135-139.
10.1007/s11011-011-9242-1
Lauridsen MM, Gronbaek H, Naeser EB, Leth ST, Vilstrup H (2012) Gender and age effects on the continuous reaction times method in volunteers and patients with cirrhosis. Metab Brain Dis. 10.1007/s11011-012-9318-6
Les I, Doval E, Garcia-Martinez R, et al. (2011) Effects of branched-chain amino acids supplementation in patients with cirrhosis and a previous episode of hepatic encephalopathy: A randomized study. Am J Gastroenterol.106:1081-1088.
ajg20119 [pii]
10.1038/ajg.2011.9
Marchesini G, Marzocchi R, Noia M, Bianchi G (2005) Branched-chain amino acid supplementation in patients with liver diseases. J Nutr.135:1596S-1601S.
135/6/1596S [pii]
Prasad S, Dhiman RK, Duseja A, Chawla YK, Sharma A, Agarwal R (2007) Lactulose improves cognitive functions and health-related quality of life in patients with cirrhosis who have minimal hepatic encephalopathy. Hepatology.45:549-559.
10.1002/hep.21533
Renzi D (1965) The comparative efficiency of intelligence and vigilance tests in detecting hemispheric cerebral damage. Cortex.1:410-433.

Romero-Gomez M, Cordoba J, Jover R, et al. (2007) Value of the critical flicker frequency in patients with minimal hepatic encephalopathy. Hepatology.45:879-885.
10.1002/hep.21586
Romero-Gomez M (2010) Pharmacotherapy of hepatic encephalopathy in cirrhosis. Expert Opin Pharmacother.11:1317-1327.
10.1517/14656561003724721
Sanyal A, Younossi ZM, Bass NM, et al. (2011) Randomised clinical trial: Rifaximin improves health-related quality of life in cirrhotic patients with hepatic encephalopathy - a double-blind placebo-controlled study. Aliment Pharmacol Ther.34:853-861.
10.1111/j.1365-2036.2011.04808.x
Sharma P, Sharma BC, Puri V, Sarin SK (2007) Critical flicker frequency: Diagnostic tool for minimal hepatic encephalopathy. J Hepatol.47:67-73.
S0168-8278(07)00145-6 [pii]
10.1016/j.jhep.2007.02.022
Sharma P, Sharma BC, Sarin SK (2010) Critical flicker frequency for diagnosis and assessment of recovery from minimal hepatic encephalopathy in patients with cirrhosis. Hepatobiliary Pancreat Dis Int.9:27-32.

1314 [pii]
Sharma P, Sharma BC, Agrawal A, Sarin SK (2012) Primary prophylaxis of overt hepatic encephalopathy in patients with cirrhosis: An open labeled randomized controlled trial of lactulose versus no lactulose. J Gastroenterol Hepatol. 10.1111/j.1440-1746.2012.07186.x
Sidhu SS, Goyal O, Mishra BP, Sood A, Chhina RS, Soni RK (2011) Rifaximin improves psychometric performance and health-related quality of life in patients with minimal hepatic encephalopathy (the rime trial). Am J Gastroenterol.106:307-316.
ajg2010455 [pii]
10.1038/ajg.2010.455
Thompson JR (2010) Treatment guidelines for hepatic encephalopathy. Pharmacotherapy.30:4S-9S.
10.1592/phco.30.pt2.4S
10.1592/phco.30.pt2.4S [pii]
Weissenborn K (2001) Neuropsychological characterization of hepatic encephalopathy. . J Hepatol.34:768-773.

Zullo A, Hassan C, Lorenzetti R (2011) Rifaximin therapy in minimal hepatic encephalopathy cirrhotics. Am J Gastroenterol.106:2041; author reply 2041-2042.
ajg2011216 [pii]
10.1038/ajg.2011.216
